# Autophagy protects HUVECs against ER stress-mediated apoptosis under simulated microgravity

**DOI:** 10.1007/s10495-019-01560-w

**Published:** 2019-07-29

**Authors:** Cheng-Fei Li, Yi-Kai Pan, Yuan Gao, Fei Shi, Yong-Chun Wang, Xi-Qing Sun

**Affiliations:** 1 Department of Aerospace Biodynamics, School of Aerospace Medicine, Fourth Military Medical University, 169 Chang Le Xi Road, Xi’an, 710032 China; 2 Key Lab of Aerospace Medicine, Chinese Ministry of Education, Xi’an, 710032 Shaanxi China

**Keywords:** Clinorotation, HUVECs, Autophagy, Apoptosis, Endoplasmic reticulum stress, Unfolded protein response

## Abstract

Astronauts exposed to a gravity-free environment experience cardiovascular deconditioning that causes post-spaceflight orthostatic intolerance and other pathological conditions. Endothelial dysfunction is an important factor responsible for this alteration. Our previous study showed enhanced autophagy in endothelial cells under simulated microgravity. The present study explored the cytoprotective role of autophagy under microgravity in human umbilical vein endothelial cells (HUVECs). We found that clinorotation for 48 h induced apoptosis and endoplasmic reticulum (ER) stress in HUVECs. ER stress and the unfolded protein response (UPR) partially contributed to apoptosis under clinorotation. Autophagy partially reduced ER stress and restored UPR signaling by autophagic clearance of ubiquitin-protein aggregates, thereby reducing apoptosis. In addition, the ER stress antagonist 4-phenylbutyric acid upregulated autophagy in HUVECs. Taken together, these findings indicate that autophagy plays a protective role against apoptosis under clinorotation by clearing protein aggregates and partially restoring the UPR.

## Introduction

The cardiovascular consequences of exposure to microgravity are evident in the form of orthostatic intolerance, reduced aerobic exercise capacity, and hypovolemia. Endothelial cells (ECs) form the inner layer of blood vessels, and their compromised function is hypothesized to be an important underlying mechanism of numerous cardiovascular diseases [1]. As highly dynamic tissue, the vascular endothelium is highly sensitive to mechanical forces and therefore undergoes significant morphological and functional changes at zero gravity via mechanotransduction processes [2], which are important causes of cardiovascular deconditioning following spaceflight.

Autophagy is an essential process for cellular homeostasis by releasing energy substrates and eliminating defective or damaged organelles. It consists of sequestration of cytoplasmic organelles and proteins within an isolation membrane followed by selective degradation [3]. Recent studies have provided evidence suggesting that simulated microgravity using random positioning machines (RPMs) activates autophagy in RAW264.7 pre-osteoclast cells and seminoma cells [4, 5]. Our previous study showed that clinorotation for 48 h increased the level of autophagy in HUVECs via the HDM2-p53-mTOR pathway [6]. However, the role of autophagy in cell adaptation to microgravity is poorly understood.

Accruing evidence has shown that there is no clear-cut distinction between apoptosis and autophagy. In fact, multiple direct and indirect interactions between apoptosis and autophagy have been described, indicating a mechanistic overlap and interaction between the two processes. Autophagy is enhanced by many stressors, such as starvation and pathogens associated with infection, leading to restoration of homeostasis. However, if the stress persists, prolonged autophagy may be deleterious and activate apoptosis [7]. In addition, the two pathways are regulated by many common factors such as B-cell lymphoma 2 (Bcl-2) and p53 [8–10]. Other proteins essential for initiation of autophagy include the class III phosphatidylinositol 3 kinase (PIK3C3)–Beclin 1 complex, which serves as a regulator of apoptosis [11]. To the best of our knowledge, whether clinostat-simulated microgravity enhances apoptosis and the interplay between autophagy and apoptosis under simulated microgravity in human umbilical vein endothelial cells (HUVECs) have not been reported.

Endoplasmic reticulum (ER) stress is triggered by loss of homeostasis in the ER, resulting in accumulation of misfolded proteins and activation of the unfolded protein response (UPR), which can increase the protein folding capacity of the ER [12]. The UPR is comprised of three classical signaling cascades, inositol requiring element 1 (IRE1), protein kinase RNA-like ER kinase (PERK), and activating transcription factor 6 (ATF6), all of which remain inactive under nonstress conditions by association with the glucose-regulated protein 78 (GRP78) ER stress marker. A recent study showed that clinostat rotation significantly increased the expression of GRP78, IRE1, and phosphorylated PERK proteins [13]. Another study used a tail suspension model to show that GRP78 in the kidney is upregulated by simulated microgravity [14]. In recent years, more light has been shed on the link between the UPR and autophagy. It has been shown that the autophagic clearance of protein aggregates inhibits neuronal death [15] and also promotes cell survival in β-cells under ER stress [16]. Despite extensive characterization of the UPR under microgravity, it is unknown whether clinorotation enhances the UPR in HUVECs, and whether autophagy induced by clinorotation counterbalances ER stress under microgravity.

The objectives of this study were to investigate the interplay between autophagy and apoptosis under microgravity and the underlying mechanisms. Our results showed that clinostat-simulated microgravity induced ER stress and the UPR, and autophagy induced by clinorotation inhibited apoptosis by blocking ER stress.

## Materials and methods

### Cell culture and drug treatment

HUVECs were obtained from the American Type Culture Collection (Manassas, VA, USA) and cultured in RPMI 1640 medium (Invitrogen, Carlsbad, CA, USA) supplemented with 10% fetal bovine serum (Invitrogen) and 1% penicillin–streptomycin at 37 °C and 5% CO_2_ in a humidified incubator. Rapamycin (APExBIO Technology, Houston, TX, USA) was dissolved in dimethyl sulfoxide (DMSO) at 5 mM for storage at − 20 °C. The autophagy inhibitor 3-methyladenine (3-MA; MP Biomedicals, Santa Ana, CA, USA) was dissolved in ddH_2_O at a concentration of 200 mM for storage at room temperature, and bafilomycin A1 (BafA1, Abcam, Cambridge, UK) was dissolved in DMSO at a concentration of 400 mM and kept at − 20 °C. The ER stress antagonist 4-phenylbutyric acid (4-PBA; Yuanye Bio-Technology, Shanghai, China) was dissolved in phosphate-buffered saline (PBS) at a concentration of 500 mM for storage at − 20 °C. The final concentrations of the drugs and duration of treatments are indicated in the figure legends.

### Simulated microgravity

Microgravity conditions were simulated using a two-dimensional clinostat. Similar changes in the cytoskeletal structure have been observed both during spaceflight and under clinorotation [17]. Our previous results documenting functional alterations in HUVECs cultured in the clinostat mimicked the results obtained in true microgravity, suggesting validity of this system for simulating the effects of microgravity [18, 19]. In this study, HUVECs were seeded at a density of 1 × 10^5^ cells per well in 6-well plates, each of which contained a coverslip 2.55 × 2.15 cm in size. Then the cells were cultured in an incubator before the coverslips were placed into the fixture of the chambers (Astronaut Research and Training Center, Beijing, China), which were subsequently filled with culture medium. Air bubbles were removed to prevent the effects of shear stress. Then the chambers rotated around the horizontal axis at 30 r/min for 48 h under no-flow conditions. The direction changes through continuous rotation are faster than the response time of cells to the gravity field and the sum of the gravitational force vectors tends to equal zero, creating effects on cells that are similar to actual microgravity. The gravitational force of about 10^−3^ g on HUVECs is simulated when the clinostat rotates at 30 r/min. The cells exposed to clinorotation were considered the microgravity group (MG), and the paralleled stationary chambers were used as the control group to eliminate the effects of other factors. The entire system was positioned in a culture incubator.

### Small interfering RNA transfection

Inhibition of autophagy was performed by knockdown of autophagy-related protein 5 (ATG5). HUVECs at 70% confluency were transfected with small interfering RNA (siRNA) targeting ATG5 (5′-CCTTTGGCCTAAGAAGAAA-3′) or siRNA-negative control (NC) with Lipofectamine 2000 (Invitrogen), according to the manufacturer’s protocol. Cells were lysed for protein expression or subjected to immunofluoresence 48 h after transfection.

### Immunofluorescence staining

Cells were fixed in 4% paraformaldehyde in PBS for 10 min and permeabilized with 0.1% Triton X-100 in PBS for 30 min at room temperature. After blocking in 10% normal goat serum, cells were incubated with rabbit polyclonal microtubule-associated protein 1 light chain 3 (LC3) antibody (1:200; Cell Signaling Technology [CST], Danvers, MA, USA) and ubiquitin antibody (1:100; Proteintech, Rosemont, IL, USA) overnight at 4 °C followed by incubation with Cy3-labeled and Alexa Fluor 488-labeled secondary antibody (1:1000; Beyotime, Shanghai, China) for 1 h at room temperature. Nuclei were stained in the dark with DAPI. Fluorescence images were visualized using the LSM 800 confocal fluorescence microscope (Zeiss, Oberkochen, Germany).

### Western blot analysis

Cells were washed with ice-cold PBS, scraped, lysed, and sonicated in radioimmunoprecipitation (RIPA) buffer supplemented with 1 mM phenylmethylsulfonyl fluoride (Beyotime) and phosphatase inhibitor cocktail (Roche, Basel, Switzerland). The protein concentration was measured using the Pierce BCA Protein Assay Kit (Thermo Fisher Scientific, Reinach, Switzerland). The proteins were subjected to 12% sodium dodecyl sulfate–polyacrylamide gel electrophoresis and then electrotransferred to a polyvinylidene fluoride membrane (Millipore, Burlington, MA, USA) at 100 V for 2 h. The membranes were blocked in 5% non-fat milk in Tris-buffered saline containing 0.1% Tween-20 (TBST) at room temperature for 2 h. After washing with TBST, the membranes were incubated for 8 h at 4 °C with primary antibodies against the following proteins: LC3 (1:1000; CST), p62 (1:1000; Proteintech), C/EBP homologous protein (CHOP, 1:5000; Proteintech), IRE1α (1:1000; Abcam), PERK (CST), phosphorylated PERK (p-PERK, Thr981; CST), cleaved caspase 3 (1:1000; CST), Bcl-2 (1:1000; Proteintech), p-IRE1α (Ser724) (1:1000; Abcam), ATF6 (1:1000; Proteintech). GADPH (1:1000, Beyotime) antibody was used for protein loading normalization. The membranes were incubated in horseradish peroxidase-labeled goat anti rabbit secondary antibody (1:5000; Proteintech) for 1 h at room temperature. The signal was detected by enhanced chemiluminescence (Millipore). Band intensity was analyzed using Image J software.

### Flow cytometry

Annexin V-FITC/PI labelling was performed to detect apoptosis [20] according to manufacturer’s recommendations (Invitrogen). At least 10,000 cells were analysed by flow cytometry to determine the percentage of apoptotic cells. The results are presented as percentage of apoptotic cells.

### Statistical analysis

All data are presented as the means ± standard deviations (SDs) of three independent experiments, and were analyzed using SPSS (version 19.0; SPSS Inc., Chicago, IL, USA) software. One-way analysis of variance was used to compare multiple treatments. Two-tailed p-values less than 0.05 were considered statistically significant.

## Results

### Clinorotation enhances autophagy in HUVECs

**Fig. 1:**
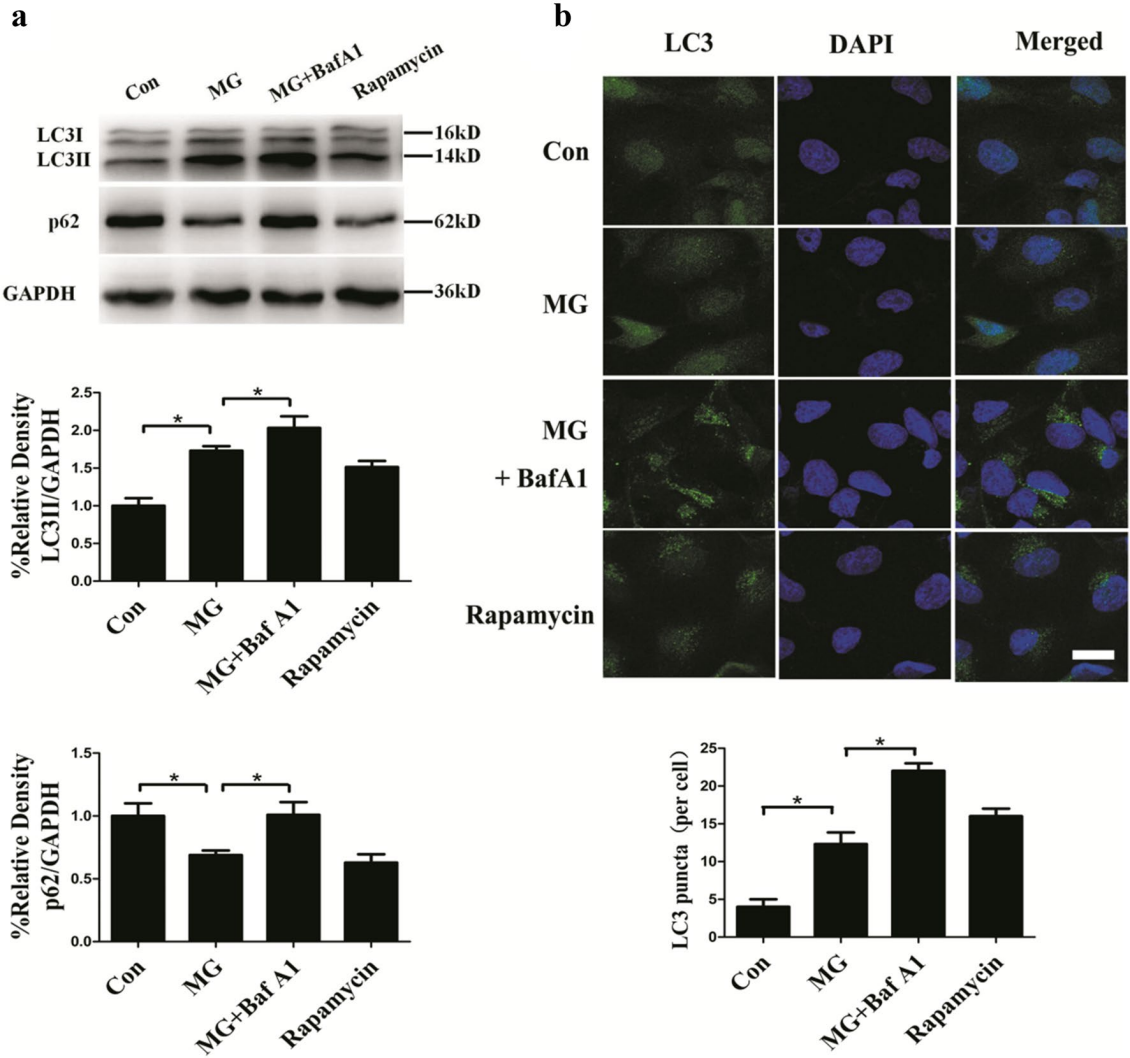
Effects of clinorotation for 48 h on autophagy level in HUVECs. **a** Western blot analysis of LC3II and p62 in HUVECs exposed to clinorotation for 48 h with or without BafA1 (20 nM) or treatment with rapamycin (1 μM) alone. **b** Immunostaining of endogenous LC3 puncta (green) and nuclei (blue) in HUVECs and the quantification of endogenous LC3 puncta per cell. The data are expressed as the mean ± SD of three replicates each. *p < 0.05 vs. the control. Scale bar: 20 μm

LC3 is initially synthesized as pro-LC3, which is processed into LC3I after the proteolysis of amino acids from the C terminus. LC3II is the final form of LC3 after LC3I conjugation to phosphatidylethanolamine when autophagy occurs. LC3II is a reliable protein marker associated with completed autophagosomes. The increase in conversion of LC3I to LC3II and the increased LC3 puncta represent autophagosome formation. Another useful marker for autophagic activity is p62, a polyubiquitin-binding protein that is degraded by autophagy; its decrease serves as an index of autophagic flux. The protein level of p62 is inversely correlated with autophagic activity [21]. As shown in Fig. 1a, HUVECs subjected to clinorotation for 48 h exhibited a marked increase of autophagic marker LC3II and decrease of p62 puncta. We also confirmed the accumulation of LC3II and p62 upon BafA1 treatment (20 nM), as BafA1 causes lysosomal damage, which impairs autophagic-lysosomal degradation [22]. Rapamycin treatment alone resulted in an increase in LC3II expression and reduction of p62. Immunofluorescence assays showed an increased number of LC3 puncta per cell under microgravity compared to the control group. Likewise, rapamycin treatment alone increased the number of LC3 puncta per cell. Moreover, treatment with BafA1 under clinorotation increased the LC3 puncta compared to the MG group (Fig. 1b), consistent with the Western blot results showing increases in endogenous LC3II, and suggesting the occurrence of autophagic flux under clinorotation. These results indicated that 48 h clinorotation activated autophagy in HUVECs.

### Autophagy inhibits apoptosis induced by microgravity in HUVECs

**Fig. 2:**
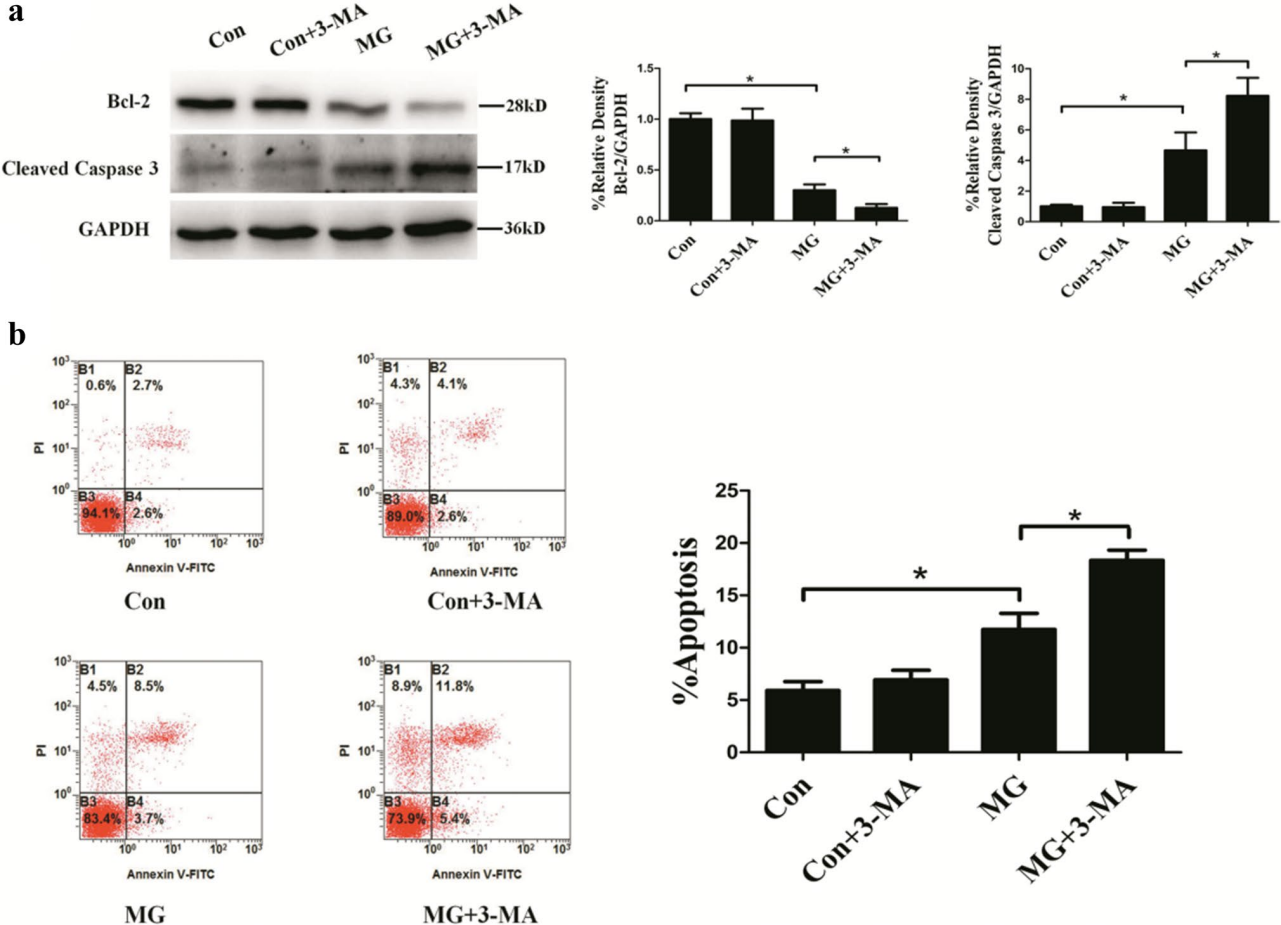
Effects of 3-MA on apoptosis rate after clinorotation for 48 h in HUVECs. **a** Western blot analysis of Bcl-2 and cleaved caspase 3 after clinorotation for 48 h with or without 3-MA (5 mM). **b** Annexin V-FITC/PI staining analyzed by flow cytometry for the percentage of apoptosis in HUVECs after 48 h clinorotation with or without 3-MA (5 mM). The data are expressed as the mean ± SD of three replicates.*p < 0.05 vs. the control

To evaluate the relationship between autophagy and apoptosis, the autophagy inhibitor 3-MA, which can block class III PI3K and thus inhibit the initiation of autophagy, was used. As shown in Fig. 2a, clinostat-simulated microgravity for 48 h in HUVECs resulted in a considerable increase in cleaved caspase 3 protein levels and decrease in Bcl-2 protein levels compared to the control group; 3-MA made these changes more apparent as detected by Western blotting. Likewise, for 48 h increased the rate of apoptosis in HUVECs, which was further increased by treatment with 3-MA compared to the MG group (Fig. 2b). Together, these results indicated that autophagy inhibited apoptosis in HUVECs under clinostat-simulated microgravity.

### Clinorotation induces ER stress and UPR in HUVECs

**Fig. 3:**
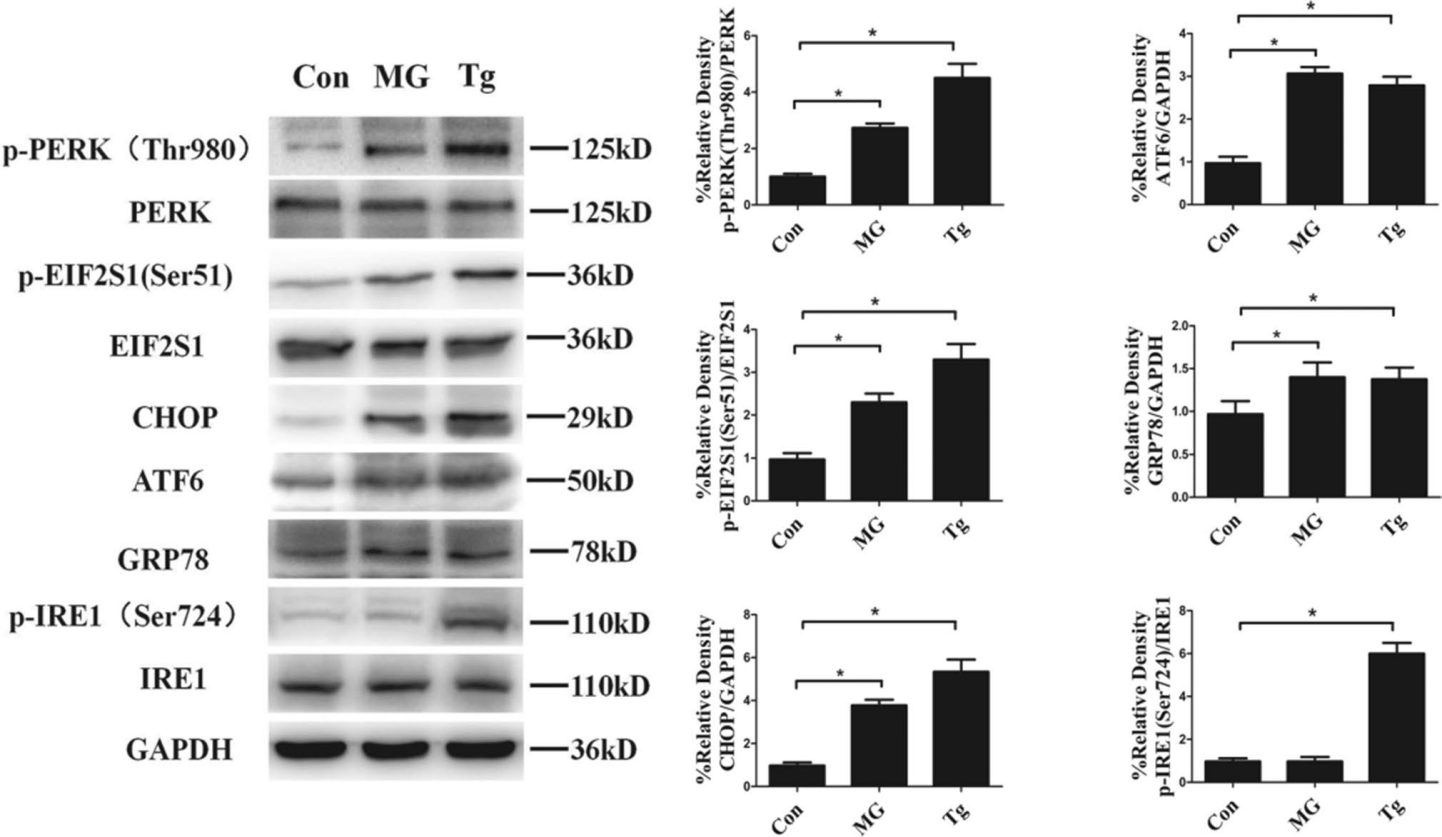
Expression of ER stress and UPR markers in HUVECs exposed to clinorotation for 48 h. Western blot analysis of p-PERK (Thr980), p-EIF2S1 (Ser51), CHOP, ATF6, GRP78 and p-IRE (Ser724) under clinorotation for 48 h or treated with Tg for 9 h at a concentration of 200 μM. The data are expressed as the mean ± SD of three replicates. *p < 0.05 vs. the control

Based on the fact that long-lasting ER stress causes cell death [15] and simulated microgravity induces ER stress in certain types of cells [13, 14], we explored three types of ER stress sensor proteins: IRE1, PERK and ATF6. The results showed that clinorotation for 48 h significantly increased the phosphorylation of PERK and its downstream protein p-eIF2S1, but had no marked effect on p-IRE1 (Fig. 3). GRP78 is another marker of ER stress and UPR. Once ER stress is activated, ATF6 is cleaved by Golgi-localized proteases, resulting in a 50 kDa cytosolic fragment. The cleaved ATF6 fragment activates the transcription of GRP78. We, therefore, monitored UPR activation by visualizing the expression of GRP78, and observed the increased expression of GRP78 after clinorotation for 48 h in HUVECs. CHOP is one of the components of the ER stress-mediated apoptosis pathway. The activation of PERK increases phosphorylation of eIF2, which induces the expression of CHOP. In addition, ATF6 and IRE1 can induce CHOP expression [23]. The expression of CHOP was also increased after clinorotation for 48 h in HUVECs. The results indicated that clinostat-simulated microgravity for 48 h caused ER stress and activated the UPR in HUVECs. Moreover, treatment with thapsigargin (Tg, 200 μM, 9 h), an ER stressor, activated IRE1, PERK, and ATF6 and increased the expression of GRP78 and CHOP.

### ER stress inhibitor 4-PBA induces autophagy in HUVECs

**Fig. 4:**
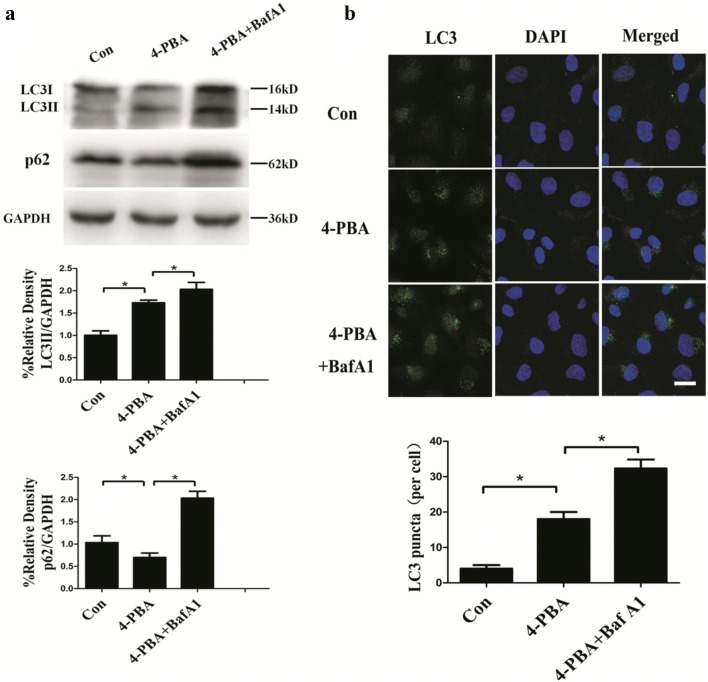
Effects of 4-PBA on autophagy level in HUVECs. **a** Western blot analysis of LC3II and p62 in HUVECs treated with 4-PBA (5 mM, 3 h) in the presence or absence of BafA1 (20 nM). **b** Immunostaining of endogenous LC3 puncta (green) and nuclei (blue) in HUVECs and quantification of endogenous LC3 puncta per cell. The data are expressed as the mean ± SD of three replicates each. *p < 0.05 vs. the control. Scale bar: 20 μm

Prolonged activation of ER stress induces apoptosis in cells under certain physiologic and pathophysiologic conditions [24]. CHOP and IRE1 branches along with subsequent activation of caspase 3 play central roles in apoptosis induced by ER stress [25, 26]. To confirm whether the induction of apoptosis was due to ER stress under microgravity in HUVECs, 4-PBA, a short-chain fatty acid chemical chaperone that improves the capacity of ER folding and reduces ER stress [27] was used to inhibit ER stress in this study. Although previous studies have revealed that 4-PBA enhances autophagy in fibroblasts and hepatocyte cells [28, 29], it remains unknown whether 4-PBA has a relationship with autophagy in HUVECs. In our study, 4-PBA treatment (5 mM, 3 h) increased LC3II protein levels and decreased p62 protein levels (Fig. 4a). The increased LC3II levels under BafA1 treatment indicated the increased flux of autophagy in 4-PBA-treated cells. The number of LC3 puncta significantly increased with 4-PBA treatment compared to the controls, and further accumulated with BafA1 treatment (Fig. 4b). These data confirmed that autophagy was activated in HUVECs treated with 4-PBA.

### ER stress mediates apoptosis under clinorotation in HUVECs

**Fig. 5:**
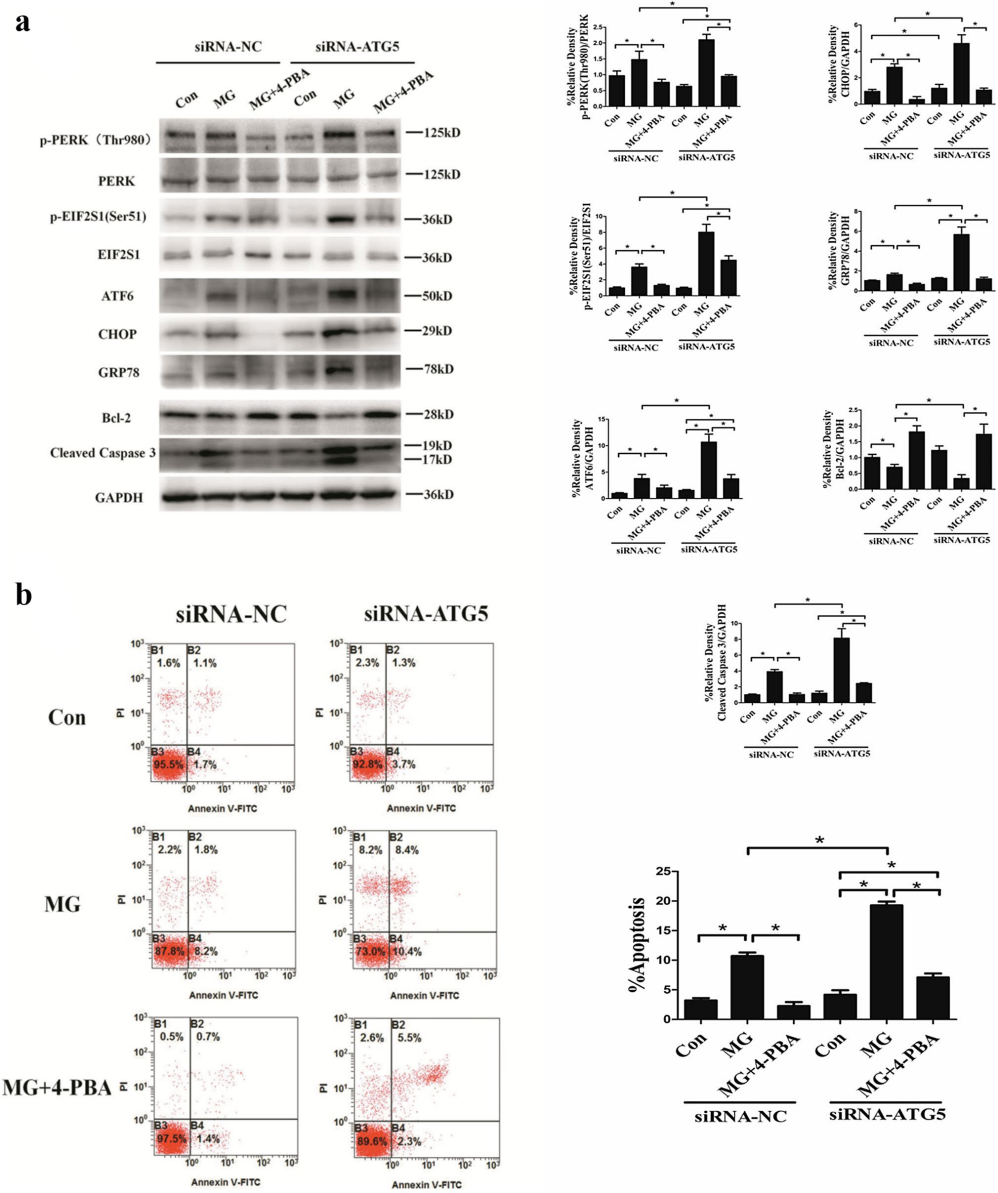
Effects of 4-PBA on apoptosis in HUVECs. **a** Western blot analysis of p-PERK (Thr980), p-EIF2S1 (Ser51), CHOP, ATF6, and GRP78, along with Bcl-2 and cleaved caspase 3 in HUVECs under 48 h clinorotation transfected with siRNA-ATG5 or siRNA-NC, alone or in combination with 4-PBA (5 mM). **b** Annexin V-FITC/PI staining for the apoptosis rates in HUVECs analyzed by flow cytometry under 48 h clinorotation transfected with siRNA-ATG5 or siRNA-NC alone or in combination with 4-PBA (5 mM). The data are expressed as the mean ± SD of three replicates each. *p < 0.05 vs. the control

The aforementioned results showed that 4-PBA enhanced autophagy in HUVECs. To evaluate the effects of ER stress on the apoptotic rate under microgravity, the ER stress inhibitor 4-PBA was used on condition that the autophagy induced by 4-PBA was ruled out. HUVECs were transfected with ATG5 siRNA to inhibit autophagy. As shown in Fig. 5a, under the condition of simulated microgravity, inhibition of autophagy with ATG5 siRNA aggravated ER stress and the UPR, as evidenced by increased phosphorylation of PERK and EIF2S1 along with the increased expression of ATF, CHOP, and GRP78 compared to MG group without ATG5 knockdown. Moreover, compared to the MG group without ATG5 knockdown, Bcl-2 was significantly decreased and cleaved caspase 3 was significantly increased in HUVECs with knockdown of ATG5 under clinorotation (Fig. 5a). Furthermore, in cells transfected with ATG5 siRNA, as expected, clinorotation induced the upregulation of ER stress markers compared with control cells, whereas 4-PBA (5 mM) prevented the upregulation of these markers and cleaved caspase 3 (apoptotic marker) and downregulated of Bcl-2. As shown in Fig. 5b, flow cytometry analysis of Annexin V-FITC/PI showed that in HUVECs under clinorotation, ATG5 knockdown increased the apoptosis rate. Moreover in agreement with the results shown above, in ATG5 knockdown cells, 4-PBA significantly inhibited apoptosis under clinorotation although it did not fully abrogate apoptosis induction by clinorotation. Collectively, these data showed that ER stress induced apoptosis under clinorotation in HUVECs.

### Autophagy protects from ER stress-mediated apoptosis in HUVECs under clinorotation

**Fig. 6:**
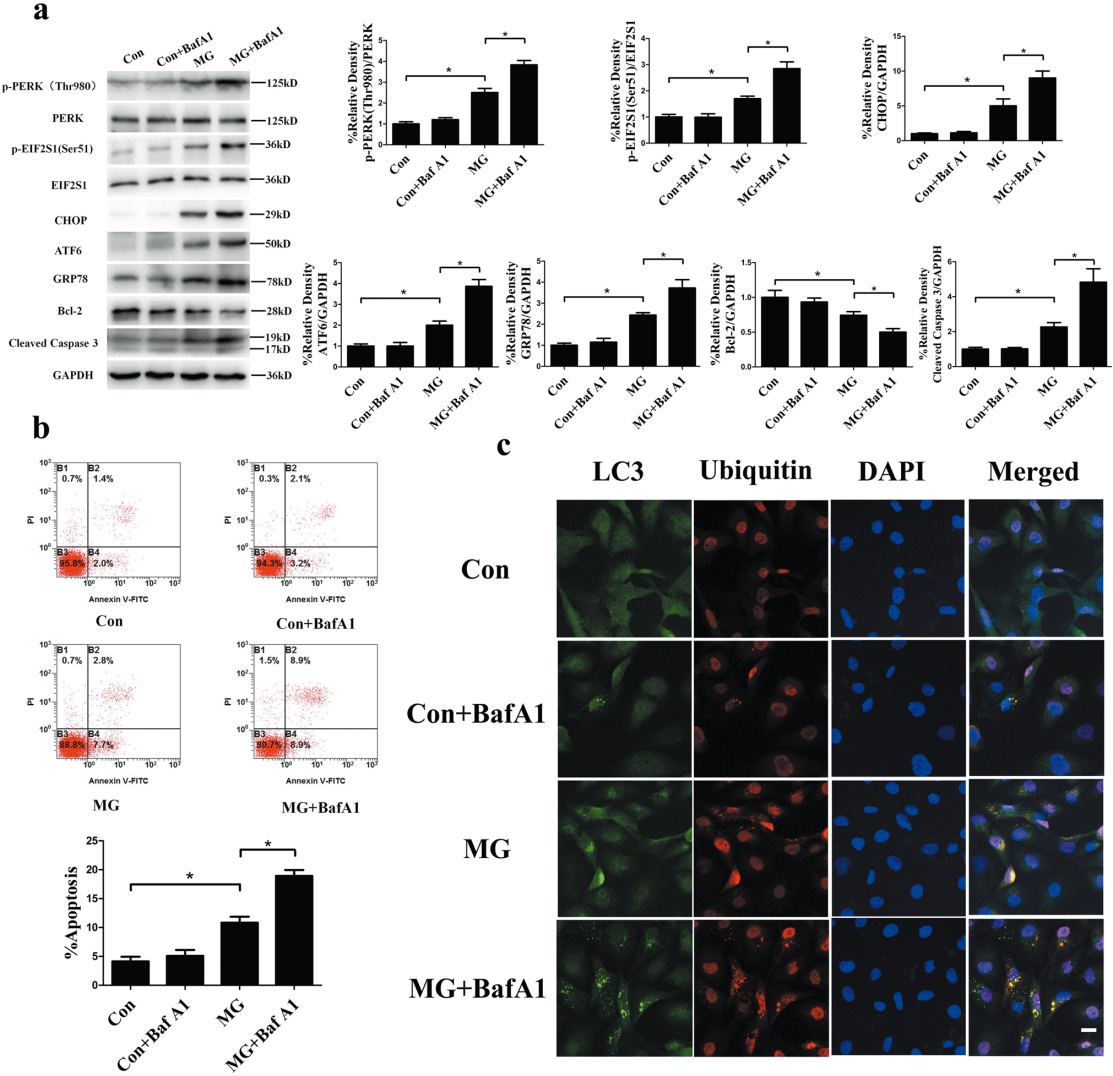
Effects of BafA1 on the UPR and ER stress-mediated apoptosis in HUVECs under clinorotation. **a** Western blot analysis of p-PERK (Thr980), p-EIF2S1 (Ser51), CHOP, ATF6, and GRP78, along with Bcl-2 and cleaved caspase 3 in HUVECs under 48 h clinorotation in the presence or absence of BafA1 (20 nM). **b** Annexin V-FITC/PI staining for the apoptosis rates in HUVECs analyzed by flow cytometry under 48 h clinorotation in the presence or absence of BafA1 (20 nM). **c** Immunostaining of endogenous LC3 puncta (green), ubiquitin (red), and nuclei (blue) in HUVECs under 48 h clinorotation in the presence or absence of BafA1 (20 nM). The data are expressed as the mean ± SD of three replicates each. *p < 0.05 vs. the control. Scale bar: 20 μm

As indicated in the aforementioned results, ER stress was responsible for the apoptosis induced by clinorotation and ER stress-induced apoptosis could be blocked, at least in part, by autophagy. It is well established that autophagy plays a protective role in ER stress-mediated apoptosis in many experimental conditions [15, 16]. Therefore, we examined whether autophagy inhibits apoptosis by alleviating ER stress and the UPR. We inactivated autophagy with BafA1 treatment and observed that treatment with BafA1 under microgravity increased protein levels of p-PERK, p-EIF2S1, ATF6, CHOP, and GRP78 compared to that for nontreated cells under clinorotation (Fig. 6a). In addition, under simulated microgravity, the BafA1-treated cells revealed increased cleaved caspase 3 and decreased Bcl-2 levels compared with untreated HUVECs (Fig. 6a). Moreover, consistent with the results above, the blockage of autophagy using BafA1 (20 nM) caused marked cell death, as confirmed by flow cytometry analysis of Annexin V-FITC/PI under clinorotation (Fig. 6b). The results indicated that inhibition of autophagy aggravated ER stress and the UPR and enhanced apoptosis in HUVECs under clinorotation. Next, we explored the interplay among autophagy, ER stress, and apoptosis. A previous study reported that autophagy protects cell from ER stress-mediated cell death by eliminating mutant-aggregated procollagen [26]. The autophagic response to ER stress induced by insulin secretion deficiency plays a pro-survival role by clearing polyubiquitinated protein aggregates in pancreatic β cells [16]. Therefore, we determined whether ubiquitin-protein was co-localized with LC3 puncta under clinorotation in HUVECs. Ubiquitinated protein within cells significantly increased after clinorotation, and lysosome marker LC3 puncta positively co-localized with ubiquitin after clinorotation for 48 h (Fig. 6c). BafA1 (20 nM) treatment inhibited autophagic clearance of ubiquitinated proteins, causing increased LC3 puncta and co-localization of ubiquitinated protein with LC3, as confirmed by immunofluorescence staining. Taken together, our present results suggested that autophagy induced by clinorotation protected HUVECs against apoptosis through clearance of unfolded ubiquitinated proteins and alleviation of ER stress.

## Discussion

In this study, we showed that clinostat-simulated microgravity enhanced autophagy levels and stimulated the UPR as an ER stressor. Autophagy induced by clinorotation had antiapoptotic effects due to autophagic clearance of ubiquitinated protein aggregates and the prevention of ER stress-mediated UPR.

ECs play a key role in cardiovascular regulation, which maintain the integrity of the vessel wall as a permeability barrier and control vascular tone [30]. ECs are very sensitive to mechanical forces, to which they respond with changes in gene expression and signaling pathways [31]. Morphological changes such as cytoskeletal remodeling [32] and functional changes in terms of proliferation [33], migration [34], and nitric oxide release [5] have been observed in ECs under microgravity. Microgravity, as a mechanical stress, increases LC3II expression in various cells [4, 5]. However, it is still difficult to ascertain autophagy enhancement under microgravity because of the lack of autophagy flux detection. In this study, the expression of LC3 and p62 was examined with BafA1, and the results revealed that clinorotation induced autophagosome formation and activated autophagy flux in HUVECs. Paradoxically, a recent study reported that colorectal cancer cells react to change in gravitational stress with reduced cell growth and increased autophagosome formation, whereas autophagy flux is downregulated [35]. Because ECs are more sensitive to microgravity than cancer cells, it is likely that there are differences in reaction to microgravity in different cell lines and rotating cell culture systems.

Both morphological and biochemical signs of apoptosis in some types of cells have been observed under microgravity. The radiation and microgravity during spaceflight were shown to alter the expression of 17 miRNAs are involved in apoptosis in *Caenorhabditis* *elegans* [36]. In addition, a previous study showed that the expression of cleaved caspase 3 and the number of apoptotic TCam-2 cells significantly increased in RPM-cultured samples after 48 h of culture [37]. Apoptosis is one of the effects of microgravity on ECs [38]. Increased apoptosis is found in pulmonary microvascular ECs after clinorotation for 72 h, and the PI3K/Akt pathway is involved in this effect [39]. The increased expression of Bax and Fas ligand, along with cell shrinkage and convolution of the nuclear outline, typical features of apoptosis, are found in porcine aortic endothelial cells exposed to hypogravity using RPM [40]. Consistently, we also found increased cleaved caspase 3 levels in HUVECs exposed to clinorotation for 48 h.

The crosstalk between apoptosis, which invariably leads to cell death, and autophagy, which has pro-survival functions, is complex. Sharing of intermediary proteins such as Beclin-1 and Bcl-2 is an important factor linking autophagy and apoptosis [41]. On one hand, autophagy is both death-inducing and survival-promoting. On the other, activation or inactivation of apoptosis can influence autophagy activation [42–44]. The findings herein showed an increase in apoptosis rate in response to clinorotation, which increased even more in the presence of 3-MA or BafA1, indicating the pro-survival function of autophagy under clinorotation in HUVECs. This conclusion is in accordance with data from previous report showing that autophagy is protective against pro-apoptotic insults [45].

ER stress and the UPR in ECs can be mediated through the mechanosensitive pathway. Disturbed flow and low magnitude shear stress act through ER stress to promote vascular cell adhesion molecule 1 expression and monocyte adhesion to the endothelium, early events in atherosclerosis [46, 47]. In this study, we found that clinorotation for 48 h significantly upregulated ER stress markers PERK and ATF6, as well as GRP78 in HUVECs, consistent with a previous study in preosteoblast cells under clinorotation [13]. A previous study demonstrated that shear stress is mechanotransduced into ER stress signals through microtubule-associated proteins and molecular motors that bind proteins on the ER membrane [48]. In light of a previous study showing that microgravity changes the cytoskeleton [32], the results of this study suggest that microgravity might induce ER stress by influencing the cytoskeleton, although the detailed underlying mechanisms need additional studies. Moreover, it is noteworthy that the effect of clinorotation on the activation of IRE1 was less pronounced compared to the other two UPR signaling pathways. This may be due to our model of persistent exposure to simulated microgravity for 48 h. IRE1 is activated shortly after ER insult, and the activation is subsequently attenuated during persistent ER stress, whereas PERK signaling is maintained throughout the ER stress [28].

Mild ER stress is protective. However, in the event of prolonged or severe ER stress, the UPR switches from cytoprotection to the initiation of apoptosis. Apoptosis induced by non-resolving chronic ER stress contributes to a number of diseases such as neurodegenerative diseases, diabetes, atherosclerosis, and renal disease [49]. Although prolonged ER stress is proapoptotic [25], the functional roles of ER stress and UPR in the apoptosis under microgravity remain largely unknown. To investigate the proapoptotic role of ER stress in HUVECs under clinorotation, the ER stress antagonist 4-PBA was used. It is worth noting that 4-PBA is closely related to autophagy, and that its role in autophagy has been the subject of debate. For example, 4-PBA attenuates lipid accumulation via autophagy in palmitate-stimulated cells [29], whereas 4-PBA reduces autophagy in osteoclasts [50] and airway epithelial cells [51]. We demonstrated here that 4-PBA enhanced autophagy flux in HUVECs. Then ATG5 was silenced to rule out the effect of 4-PBA on autophagy due to the close relationship between autophagy and apoptosis. Our results showed that 4-PBA improved cell survival in the presence of ATG5 siRNA under microgravity conditions, indicating the crucial role of ER stress in apoptosis under clinorotation. Many reports have concluded that apoptosis occurs under microgravity through different signaling ways. A recent report showed that miR-503-p induced apoptosis in HPMECs under simulated microgravity through inhibition of Bcl-2 [52]. Simulated microgravity enhances cell apoptosis through the mTORC1/NF-κB and ERK1/2 pathways in B-cell lymphoma 6 cells [53]. Our findings demonstrated, for the first time, that ER stress induced the apoptosis of HUVECs under simulated microgravity. However, 4-PBA with ATG5 siRNA could not completely eliminate apoptotic death induced by clinorotation, indicating that other apoptotic pathways in HUVECS with a role should be further studied.

Much is known about the anti-apoptotic effects of autophagy, but the mechanism by which autophagy suppress apoptosis has remained unclear. During the degradative process, engulfed cell cargo is degraded to conserve energy and restore homeostasis. In addition, autophagy prevents activation of the apoptotic pathway by degrading impaired mitochondria or pro-apoptotic factors such as caspases [54]. ER stress featuring aberrant aggregates of misfolded proteins can be reduced by protein degradation mainly through the ER-associated degradation pathway and macroautophagy [55]. Therefore, counterbalancing ER stress during the UPR may also explain the antiapoptotic role of autophagy apart from the roles described above. The autophagic elimination of mutant-aggregated procollagen protects cell against ER stressors [23]. Autophagy protects against cell death caused by ER stress possibly by degrading unfolded proteins in neuroblastoma cells [56]. In pancreatic β-cells under ER stress conditions, autophagy inhibits cell death through clearance of protein aggregates [16]. In agreement with these findings, we found that ubiquitinated proteins aggregated in HUVECs under clinorotation and BafA1 blocked autophagic clearance of protein aggregates and increased the apoptosis rate. Recently, several mechanisms underlying apoptosis induced by accumulation of ubiquitinated proteins under ER stress have been proposed. One mechanism involves CHOP and IRE1 as discussed above. Another mechanism is the accumulation of pro-apoptotic proteins such as IκB [57], death receptor-5 [58], and Bcl-2 ovarian killer [59], which rely on degradation by the proteasome to maintain cell stability. Our study revealed that autophagy inhibition increased the unfolded protein burden, resulting in increased expression of CHOP. Whether the latter mechanism is involved in apoptosis induction by autophagy inhibition remains to be further elucidated. Our data suggest that autophagy attenuates apoptosis by degrading protein aggregates in HUVECs under simulated microgravity. Interestingly, the increased protein expression of heat shock protein 70 (Hsp70) protects HUVECs under microgravity from apoptosis [60]. Hsp70 as a molecular chaperone is closely related to ER stress, because it can facilitate the folding of proteins under stress and GRP78 is an ER chaperone protein belonging to the Hsp70 family [61]. The heat shock protein chaperone system and autophagy are two major intracellular homeostatic systems [62]. Therefore, whether Hsp70 inhibits apoptosis by alleviating ER stress and the relationship between autophagy and Hsp70 under clinorotation in HUVECs should be studied in the future. In addition, it is noteworthy that our conclusion obtained from simulated microgravity should be compared with data obtained in real spaceflight because of differences in the experimental conditions [32, 63].

In summary, this study demonstrated that ER stress and the UPR induced after clinorotation for 48 h accounted for, at least in part, the increased apoptosis of HUVECs. Clinorotation-mediated autophagy activation increased the clearance of ubiquitinated protein aggregates, thus protecting cells against apoptosis. With further studies on this topic, autophagy activators or UPR blockers could be designed to decrease the apoptosis of vascular ECs during the process of cell adaption to microgravity, and might counter severe cardiovascular deconditioning. Moreover, adaption to microgravity including cardiovascular deconditioning in real spaceflight is similar to age-related deconditioning [64], so these results may facilitate a greater understanding of common diseases.

## Abbreviations

HUVECs: Human umbilical vein endothelial cells
ER: Endoplasmic reticulum
UPR: Unfolded protein response
ECs: Endothelial cells
RPMs: Random positioning machines
Bcl-2: B cell lymphoma 2
PIK3C3: Class III phosphatidylinositol 3 kinase
IRE1: Inositol requiring element 1
DMSO: Dimethyl sulfoxide
3-MA: 3-Methyladenine
BafA1: Bafilomycin A1
4-PBA: 4-Phenylbutyric acid
PERK: Protein kinase RNA-like ER kinase
ATF6: Activating transcription factor 6
GRP78: Glucose-regulated protein 78
LC3: Microtubule-associated protein 1 light chain 3
CHOP: C/EBP homologous protein
Tg: Thapsigargin
mTORC1: Mammalian target of rapamycin complex 1
Hsp70: Heat shock protein 70
MG: Microgravity

## References

[1] Dorland YL, Huveneers S (2017). Cell-cell junctional mechanotransduction in endothelial remodeling. Cell Mol Life Sci.

[2] Li N, Wang C, Sun S, Zhang C, Lu D, Chen Q, Long M (2018). Microgravity-induced alterations of inflammation-related mechanotransduction in endothelial cells on board SJ-10 satellite. Front Physiol.

[3] Klionsky DJ (2007). Autophagy: from phenomenology to molecular understanding in less than a decade. Nat Rev Mol Cell Biol.

[4] Sambandam Y, Townsend MT, Pierce JJ, Lipman CM, Haque A, Bateman TA, Reddy SV (2014). Microgravity control of autophagy modulates osteoclastogenesis. Bone.

[5] Morabito C, Guarnieri S, Catizone A, Schiraldi C, Ricci G, Mariggio MA (2017). Transient increases in intracellular calcium and reactive oxygen species levels in TCam-2 cells exposed to microgravity. Sci Rep.

[6] Li CF, Sun JX, Gao Y, Shi F, Pan YK, Wang YC, Sun XQ (2018). Clinorotation-induced autophagy via HDM2-p53-mTOR pathway enhances cell migration in vascular endothelial cells. Cell Death Dis.

[7] Booth LA, Tavallai S, Hamed HA, Cruickshanks N, Dent P (2014). The role of cell signalling in the crosstalk between autophagy and apoptosis. Cell Signal.

[8] Maiuri MC, Zalckvar E, Kimchi A, Kroemer G (2007). Self-eating and self-killing: crosstalk between autophagy and apoptosis. Nat Rev Mol Cell Biol.

[9] Pattingre S, Tassa A, Qu X, Garuti R, Liang XH, Mizushima N, Packer M, Schneider MD, Levine B (2005). Bcl-2 antiapoptotic proteins inhibit Beclin 1-dependent autophagy. Cell.

[10] Oral O, Akkoc Y, Bayraktar O, Gozuacik D (2016). Physiological and pathological significance of the molecular cross-talk between autophagy and apoptosis. Histol Histopathol.

[11] Kang R, Zeh HJ, Lotze MT, Tang D (2011). The Beclin 1 network regulates autophagy and apoptosis. Cell Death Differ.

[12] Ron D, Walter P (2007). Signal integration in the endoplasmic reticulum unfolded protein response. Nat Rev Mol Cell Biol.

[13] Yoo YM, Han TY, Kim HS (2016). Melatonin suppresses autophagy induced by clinostat in preosteoblast MC3T3-E1 cells. Int J Mol Sci.

[14] Ding Y, Zou J, Li Z, Tian J, Abdelalim S, Du F, She R, Wang D, Tan C, Wang H, Chen W, Lv D, Chang L (2011). Study of histopathological and molecular changes of rat kidney under simulated weightlessness and resistance training protective effect. PLoS ONE.

[15] Fouillet A, Levet C, Virgone A, Robin M, Dourlen P, Rieusset J, Belaidi E, Ovize M, Touret M, Nataf S, Mollereau B (2012). ER stress inhibits neuronal death by promoting autophagy. Autophagy.

[16] Bachar-Wikstrom E, Wikstrom JD, Ariav Y, Tirosh B, Kaiser N, Cerasi E, Leibowitz G (2013). Stimulation of autophagy improves endoplasmic reticulum stress-induced diabetes. Diabetes.

[17] Vorselen D, Roos WH, MacKintosh FC, Wuite GJ, van Loon JJ (2014). The role of the cytoskeleton in sensing changes in gravity by nonspecialized cells. FASEB J.

[18] Shi F, Zhao TZ, Wang YC, Cao XS, Yang CB, Gao Y, Li CF, Zhao JD, Zhang S, Sun XQ (2016). The impact of simulated weightlessness on endothelium-dependent angiogenesis and the role of caveolae/caveolin-1. Cell Physiol Biochem.

[19] Wang YC, Zhang S, Du TY, Wang B, Sun XQ (2009). Clinorotation upregulates inducible nitric oxide synthase by inhibiting AP-1 activation in human umbilical vein endothelial cells. J Cell Biochem.

[20] Nowak-Sliwinska P, Alitalo K, Allen E (2018). Consensus guidelines for the use and interpretation of angiogenesis assays. Angiogenesis.

[21] Pluquet O, Qu LK, Baltzis D, Koromilas AE (2005). Endoplasmic reticulum stress accelerates p53 degradation by the cooperative actions of Hdm2 and glycogen synthase kinase 3beta. Mol Cell Biol.

[22] Mizushima N, Yoshimori T, Levine B (2010). Methods in mammalian autophagy research. Cell.

[23] Li Y, Guo Y, Tang J, Jiang J, Chen Z (2014). New insights into the roles of CHOP-induced apoptosis in ER stress. Acta Biochim Biophys Sin (Shanghai).

[24] Szegezdi E, Logue SE, Gorman AM, Samali A (2006). Mediators of endoplasmic reticulum stress-induced apoptosis. EMBO Rep.

[25] Tabas I, Ron D (2011). Integrating the mechanisms of apoptosis induced by endoplasmic reticulum stress. Nat Cell Biol.

[26] Ishida Y, Yamamoto A, Kitamura A, Lamande SR, Yoshimori T, Bateman JF, Kubota H, Nagata K (2009). Autophagic elimination of misfolded procollagen aggregates in the endoplasmic reticulum as a means of cell protection. Mol Biol Cell.

[27] Ibrahim SH, Kohli R, Gores GJ (2011). Mechanisms of lipotoxicity in NAFLD and clinical implications. J Pediatr Gastroenterol Nutr.

[28] Besio R, Iula G, Garibaldi N, Cipolla L, Sabbioneda S, Biggiogera M, Marini JC, Rossi A, Forlino A (2018). 4-PBA ameliorates cellular homeostasis in fibroblasts from osteogenesis imperfecta patients by enhancing autophagy and stimulating protein secretion. Biochim Biophys Acta Mol Basis Dis.

[29] Nissar AU, Sharma L, Mudasir MA, Nazir LA, Umar SA, Sharma PR, Vishwakarma RA, Tasduq SA (2017). Chemical chaperone 4-phenyl butyric acid (4-PBA) reduces hepatocellular lipid accumulation and lipotoxicity through induction of autophagy. J Lipid Res.

[30] Girn HR, Orsi NM, Homer-Vanniasinkam S (2007). An overview of cytokine interactions in atherosclerosis and implications for peripheral arterial disease. Vasc Med.

[31] Tzima E, Irani-Tehrani M, Kiosses WB, Dejana E, Schultz DA, Engelhardt B, Cao G, DeLisser H, Schwartz MA (2005). A mechanosensory complex that mediates the endothelial cell response to fluid shear stress. Nature.

[32] Versari S, Longinotti G, Barenghi L, Maier JA, Bradamante S (2013). The challenging environment on board the International Space Station affects endothelial cell function by triggering oxidative stress through thioredoxin interacting protein overexpression: the ESA-SPHINX experiment. FASEB J.

[33] Mariotti M, Maier JA (2008). Gravitational unloading induces an anti-angiogenic phenotype in human microvascular endothelial cells. J Cell Biochem.

[34] Siamwala JH, Majumder S, Tamilarasan KP, Muley A, Reddy SH, Kolluru GK, Sinha S, Chatterjee S (2010). Simulated microgravity promotes nitric oxide-supported angiogenesis via the iNOS-cGMP-PKG pathway in macrovascular endothelial cells. FEBS Lett.

[35] Arun RP, Sivanesan D, Vidyasekar P, Verma RS (2017). PTEN/FOXO3/AKT pathway regulates cell death and mediates morphogenetic differentiation of Colorectal Cancer Cells under simulated microgravity. Sci Rep.

[36] Gao Y, Li S, Xu D, Wang J, Sun Y (2015). Changes in apoptotic microRNA and mRNA expression profiling in caenorhabditis elegans during the Shenzhou-8 mission. J Radiat Res.

[37] Ferranti F, Caruso M, Cammarota M, Masiello MG, Corano SK, Fabrizi C, Fumagalli L, Schiraldi C, Cucina A, Catizone A, Ricci G (2014). Cytoskeleton modifications and autophagy induction in TCam-2 seminoma cells exposed to simulated microgravity. Biomed Res Int.

[38] Maier JA, Cialdai F, Monici M, Morbidelli L (2015). The impact of microgravity and hypergravity on endothelial cells. Biomed Res Int.

[39] Kang CY, Zou L, Yuan M, Wang Y, Li TZ, Zhang Y, Wang JF, Li Y, Deng XW, Liu CT (2011). Impact of simulated microgravity on microvascular endothelial cell apoptosis. Eur J Appl Physiol.

[40] Morbidelli L, Monici M, Marziliano N, Cogoli A, Fusi F, Waltenberger J, Ziche M (2005). Simulated hypogravity impairs the angiogenic response of endothelium by up-regulating apoptotic signals. Biochem Biophys Res Commun.

[41] Zambrano J, Yeh ES (2016). Autophagy and apoptotic crosstalk: mechanism of therapeutic resistance in HER2-positive breast cancer. Breast Cancer (Auckl).

[42] Maiuri MC, Le Toumelin G, Criollo A, Rain JC, Gautier F, Juin P, Tasdemir E, Pierron G, Troulinaki K, Tavernarakis N, Hickman JA, Geneste O, Kroemer G (2007). Functional and physical interaction between Bcl-X(L) and a BH3-like domain in Beclin-1. EMBO J.

[43] Luo S, Garcia-Arencibia M, Zhao R, Puri C, Toh PP, Sadiq O, Rubinsztein DC (2012). Bim inhibits autophagy by recruiting Beclin 1 to microtubules. Mol Cell.

[44] Shimizu S, Kanaseki T, Mizushima N, Mizuta T, Arakawa-Kobayashi S, Thompson CB, Tsujimoto Y (2004). Role of Bcl-2 family proteins in a non-apoptotic programmed cell death dependent on autophagy genes. Nat Cell Biol.

[45] Wang J, Qi Q, Zhou W, Feng Z, Huang B, Chen A, Zhang D, Li W, Zhang Q, Jiang Z, Bjerkvig R, Prestegarden L, Thorsen F, Wang X, Li X, Wang J (2018). Inhibition of glioma growth by flavokawain B is mediated through endoplasmic reticulum stress induced autophagy. Autophagy.

[46] Bailey KA, Haj FG, Simon SI, Passerini AG (2017). Atherosusceptible shear stress activates endoplasmic reticulum stress to promote endothelial inflammation. Sci Rep.

[47] Chung J, Kim KH, Lee SC, An SH, Kwon K (2015). Ursodeoxycholic acid (UDCA) exerts anti-atherogenic effects by inhibiting endoplasmic reticulum (ER) stress induced by disturbed flow. Mol Cells.

[48] Westrate LM, Lee JE, Prinz WA, Voeltz GK (2015). Form follows function: the importance of endoplasmic reticulum shape. Annu Rev Biochem.

[49] Kaufman RJ (2002). Orchestrating the unfolded protein response in health and disease. J Clin Invest.

[50] Park HJ, Son HJ, Sul OJ, Suh JH, Choi HS (2018). 4-Phenylbutyric acid protects against lipopolysaccharide-induced bone loss by modulating autophagy in osteoclasts. Biochem Pharmacol.

[51] He B, Chen Q, Zhou D, Wang L, Liu Z (2018). Role of reciprocal interaction between autophagy and endoplasmic reticulum stress in apoptosis of human bronchial epithelial cells induced by cigarette smoke extract. IUBMB Life.

[52] Tang NP, Hui TT, Ma J, Mei QB (2019). Effects of miR-503-5p on apoptosis of human pulmonary microvascular endothelial cells in simulated microgravity. J Cell Biochem.

[53] Zhao T, Li R, Tan X, Zhang J, Fan C, Zhao Q, Deng Y, Xu A, Lukong KE, Genth H, Xiang J (2018). Simulated microgravity reduces focal adhesions and alters cytoskeleton and nuclear positioning leading to enhanced apoptosis via suppressing FAK/RhoA-mediated mTORC1/NF-kappaB and ERK1/2 pathways. Int J Mol Sci.

[54] Narendra D, Tanaka A, Suen DF, Youle RJ (2008). Parkin is recruited selectively to impaired mitochondria and promotes their autophagy. J Cell Biol.

[55] Yang DS, Kumar A, Stavrides P, Peterson J, Peterhoff CM, Pawlik M, Levy E, Cataldo AM, Nixon RA (2008). Neuronal apoptosis and autophagy cross talk in aging PS/APP mice, a model of Alzheimer’s disease. Am J Pathol.

[56] Ogata M, Hino S-i, Saito A, Morikawa K, Kondo S, Kanemoto S, Murakami T, Taniguchi M, Tanii I, Yoshinaga K, Shiosaka S, Hammarback JA, Urano F, Imaizumi K (2006). Autophagy is activated for cell survival after endoplasmic reticulum stress. Mol Cell Biol.

[57] Nencioni A, Grunebach F, Patrone F, Ballestrero A, Brossart P (2007). Proteasome inhibitors: antitumor effects and beyond. Leukemia.

[58] Lu M, Lawrence DA, Marsters S, Acosta-Alvear D, Kimmig P, Mendez AS, Paton AW, Paton JC, Walter P, Ashkenazi A (2014). Opposing unfolded-protein-response signals converge on death receptor 5 to control apoptosis. Science.

[59] Llambi F, Wang YM, Victor B, Yang M, Schneider DM, Gingras S, Parsons MJ, Zheng JH, Brown SA, Pelletier S, Moldoveanu T, Chen T, Green DR (2016). BOK is a non-canonical BCL-2 family effector of apoptosis regulated by ER-associated degradation. Cell.

[60] Cazzaniga A, Locatelli L, Castiglioni S, Maier JAM (2019). The dynamic adaptation of primary human endothelial cells to simulated microgravity. FASEB J.

[61] Zheng XB, Xu F, Liang H, Cao HY, Cai MY, Xu W, Weng JP (2017). SIRT1/HSF1/HSP pathway is essential for exenatide-alleviated, lipid-induced hepatic endoplasmic reticulum stress. Hepatology.

[62] Dokladny K, Zuhl MN, Mandell M, Bhattacharya D, Schneider S, Deretic V, Moseley PL (2013). Regulatory coordination between two major intracellular homeostatic systems heat shock response and autophagy. J Biol Chem.

[63] Buravkova L, Romanov Y, Rykova M, Grigorieva O, Merzlikina N (2005). Cell-to-cell interactions in changed gravity: ground-based and flight experiments. Acta Astronaut.

[64] Vernikos J, Schneider VS (2010). Space, gravity and the physiology of aging: parallel or convergent disciplines? A mini-review. Gerontology.

